# Impact of vertical positioning on lung aeration among mechanically ventilated intensive care unit patients: a randomized crossover clinical trial

**DOI:** 10.5935/2965-2774.20230069-en

**Published:** 2023

**Authors:** Douglas Neves, Paulo Ricardo Marques Filho, Raquel da Silva Townsend, Cristiano dos Santos Rodrigues, Luciana Tagliari, Laura Cordeiro Madeira, Mariana Fensterseifer Mattioni, Márcio Luiz Ferreira de Camillis, Clarissa Garcia Soares Leães, Juliana Mara Stormovski de Andrade, Caroline Cabral Robinson, Daniel Sganzerla, Laura Drehmer, Denis Fernandes Madruga da Costa, André Sant’Ana Machado, Regis Goulart Rosa, Pedro Dal Lago

**Affiliations:** 1 Intensive Care Unit, Hospital Ernesto Dornelles - Porto Alegre (RS), Brazil; 2 Intensive Care Unit, Hospital Moinhos de Vento - Porto Alegre (RS), Brazil; 3 Research Projects Office, Hospital Moinhos de Vento - Porto Alegre (RS), Brazil; 4 Postgraduate Program in Rehabilitation Sciences, Universidade Federal de Ciências da Saúde de Porto Alegre - Porto Alegre (RS), Brazil

**Keywords:** Sitting position, Standing position, Lung, Aeration, Respiration, artificial, Ultrasonography, Intensive care units

## Abstract

**Objective:**

To assess the impact of different vertical positions on lung aeration in patients receiving invasive mechanical ventilation.

**Methods:**

An open-label randomized crossover clinical trial was conducted between January and July 2020. Adults receiving invasive mechanical ventilation for > 24 hours and < 7 days with hemodynamic, respiratory and neurological stability were randomly assigned at a 1:1 ratio to the sitting position followed by passive orthostasis condition or the passive orthostasis followed by the sitting position condition. The primary outcome was lung aeration assessed using the lung ultrasound score (score ranges from 0 [better] to 36 [worse]).

**Results:**

A total of 186 subjects were screened; of these subjects, 19 were enrolled (57.8% male; mean age, 73.2 years). All participants were assigned to receive at least one verticalization protocol. Passive orthostasis resulted in mean lung ultrasound scores that did not differ significantly from the sitting position (11.0 *versus* 13.7; mean difference, -2.7; [95%CI -6.1 to 0.71; p = 0.11). Adverse events occurred in three subjects in the passive orthostasis group and in one in the sitting position group (p = 0.99).

**Conclusion:**

This analysis did not find significant differences in lung aeration between the sitting and passive orthostasis groups. A randomized crossover clinical trial assessing the impact of vertical positioning on lung aeration in patients receiving invasive mechanical ventilation is feasible. Unfortunately, the study was interrupted due to the need to treat COVID-19 patients.

ClinicalTrials.gov registry: NCT04176445

## INTRODUCTION

Invasive mechanical ventilation (IMV), despite saving lives in the intensive care unit (ICU), may result in neuromuscular damage and represents a risk factor for developing ventilator-associated lung aeration/perfusion and impairment of respiratory system function.^(1,2)^ This damage can be worsened by immobilization in bed^(3)^ or reduced by using body positioning protocols.^(4)^

Body positioning is associated with lung ventilation (aeration) and perfusion changes and has positive effects on the respiratory systems of patients receiving IMV, mainly when performed outside the bed. For example, the combination of sitting in a chair and physical activity can improve lung aeration during IMV^(5)^ using an endotracheal tube. Accordingly, passive orthostasis with the support of a tilt-table has been incorporated into practice to allow body positioning of critical care patients outside the bed.^(6)^ When included in daily routines, this strategy is associated with improving the level of consciousness of ICU patients.^(7)^ Moreover, when patients receiving IMV are placed in passive orthostasis using a tilt-table, there is a transient increase in minute volume without a significant change in oxygenation.^(8)^

Despite the benefits of mobilizing patients outside the bed, evidence is still limited on the effects of vertical positioning on lung aeration, especially when vertical positioning is performed passively in the orthostatic position. Therefore, this study aimed to assess the effects of different vertical positions on lung aeration in critically ill patients receiving IMV. Based on the physiological ventilatory changes in the upright posture, we hypothesized that the verticalization of the chest would improve the pulmonary aeration of patients receiving IMV. The specific objectives were to evaluate variations in the tidal volume, respiratory rate and minute volume of patients receiving IMV and the safety of mobilizing patients outside of bed. Considering the demand on professionals to verticalize patients receiving IMV, we tried to determine the number of team members necessary to position patients receiving IMV in different positions outside of bed.

## METHODS

The present study was designed as an open-label, randomized, crossover, two-center clinical trial to assess the impact of different vertical positions on lung aeration in hospitalized ICU patients receiving IMV. Patients were enrolled from January to July 2020 and followed from the medical-surgical ICUs of *Hospital Ernesto Dornelles* (40 beds) and *Hospital Moinhos de Vento* (17 beds), which are both tertiary, academic, and private hospitals in southern Brazil. This study was approved by the institutional review boards of *Hospital Ernesto Dornelles* (approval number 3.335.370) and *Hospital Moinhos de Vento* (approval number 3.243.829). In addition, informed consent was obtained from the legally authorized representatives of all participants before study enrollment. This study was conducted according to resolution number 466/2012 of The Brazilian National Health Council and was registered at ClinicalTrials.gov (NCT04176445) before the first patient was recruited. This research did not receive specific grant from funding agencies in the public, commercial, or for-profit sectors. The study was stopped earlier than planned due to the coronavirus disease 2019 (COVID-19) pandemic. The dedication of study staff to health care activities for severely ill COVID-19 patients precluded the maintenance of study procedures.

All consecutive subjects ≥ 18 years of age admitted to the ICU and ventilated for ≥ ¬¬24 hours and ≤ 7 days, without an extubation plan on the day of the study protocols, were eligible for inclusion. The exclusion criteria were as follows: a noradrenaline level > 0.2mcg/kg/minute; a > 50% increase in the dose of noradrenaline (as long as it exceeded 0.1 mcg/kg/minute) within 2 hours prior to enrollment; a sodium nitroprusside level > 1 mcg/kg/minute; a heart rate < 40 or > 130bpm; active myocardial ischemia; a systolic blood pressure > 200mmHg or a mean arterial blood pressure < 65 mmHg; arrhythmia; the presence of an intra-aortic balloon counterpulsation; a fraction of inspired oxygen > 60%; a positive end-expiratory pressure ≥ 10cmH_2_O; a peripheral oxygen saturation < 88%; a respiratory rate < 5 or > 40bpm; a diagnosis of Acute Respiratory Distress Syndrome (ARDS); a Richmond Agitation-Sedation Scale (RASS) score < -4 or > +1; intracranial hypertension; a diagnosis of neurological and/or neuromuscular diseases that would prevent mobilization; acute spinal cord injury and/or the risk of instability; acute phase of stroke; fracture or amputation of the lower limbs; the inability to walk unaided before critical illness in the ICU (walking with the use of a cane or walker was not an exclusion); a Medical Research Council (MRC) strength scale score ≥ 3 in the lower limbs; pressure ulcer in the heel region; suspicion or confirmation of COVID-19; infusion of neuromuscular blocking agents; the presence of a peritoneostomy; extensive burns; a temperature > 38.5°C; active gastrointestinal bleeding; intra-abdominal hypertension; thrombocytopenia (a platelet count < 50,000 units/mm3); bulky diarrhea; hypoglycemia (hemoglucotest < 70mg/dL); intermittent renal replacement therapy; major abdominal surgeries; and the presence of a peridural catheter.

### Interventions

**Sitting position protocol:** participants were passively placed in bedside sedestation with back support, where they remained for 30 minutes; their hips and knees were flexed at 90°, and their feet were supported; this position aimed to simulate sitting in a chair.

**Passive orthostasis protocol:** participants were transferred to a tilt-table (0° inclination). Safety straps were placed on the knees, waist, and chest to keep the participants in the orthostatic position. The tilt-table protocol lasted 30 minutes. Initially, participants were placed in a vertical position up to 45° and remained in this position for 3 minutes. Next, they were tilted to 60°, where they remained for 2 minutes. Then, verticalization was performed up to 75 - 85°, where they remained for another 25 minutes.

**Cointerventions:** endotracheal aspiration was performed 30 minutes before the beginning of both verticalization position protocols.

According to local protocols, the critical care management of participants, including IMV parameters, was left at the discretion of each center assistant team.

### Randomization, washout, and blinding

Subjects were randomized at a 1:1 ratio to one of two verticalization groups: the sitting position followed by passive orthostasis group or the passive orthostasis followed by the sitting position group. Participants were randomized on the same day they were deemed to be suited to participate in the study. Randomization was performed using blocks of different sizes and stratified by center. Allocation sequencing and concealment were ensured through the use of a centralized web-based randomization platform (REDCap, Vanderbilt University, Nashville, TN, USA).^(9)^ Patients were screened daily by a member of the study (1 in each center) to identify those able to be included. After meeting the criteria, the study member enrolled participants on the platform for randomization. Researchers had access to the intervention sequence only after the participants were registered on the platform. A washout window period (90 to 150 minutes) in which the patient was returned to bed was applied between the two verticalization protocols to avoid the carry-over effect. Considering the nature of the trial interventions, blinding was not feasible.

### Outcomes

The primary outcome was lung aeration assessed using the lung ultrasound score (LUS) at the end of each verticalization protocol (sitting position and passive orthostasis), while the patients were in the vertical position. The LUS was also measured at 3 additional time points to assess the consistency of the findings: while in the supine position in bed (baseline), while in the supine position in bed after the sitting position, and while in the supine position in bed after passive orthostasis. For measurements while in the supine position, the subjects were placed with the headboard elevated to 30°. Lung aeration was assessed through chest ultrasound (Sonosite®), for which a convex transducer was used. The intercostal spaces of the anterior, lateral and posterior regions of both lungs were investigated. The division landmark was the anterior and posterior axillary lines, with each area being divided into upper and lower regions. Thus, six representative zones of each lung were assessed. Following the standards already established by the LUS, normal aeration was represented by pleural sliding and horizontal A-lines or by at least three vertical B-lines, and a score of 0 was assigned in this case. When a moderate loss of aeration occurred, characterized by multiple B-lines, either regularly or irregularly spaced, originating from the pleural line or small juxtapleural consolidations, a score of 1 was assigned. When coalescent B-lines were present in several intercostal spaces occupying the whole intercostal space and characterizing a severe loss of lung aeration, a score of 2 was assigned. If there was a total lung aeration loss, as observed in lung consolidation, with tissue echogenicity and static and dynamic air bronchograms, the investigated region was given a score of 3. The total LUS score was determined by summing the 12 areas examined, with scores ranging from 0 to 36; the higher the score was, the worse the lung aeration.^(10)^ The worst ultrasound abnormality detected was considered to characterize the region examined. All assessments were performed by trained individuals with clinical experience who had performed at least 100 lung ultrasound procedures.^(11)^

Secondary outcomes included the variation tidal volume (expressed in mL), respiratory rate (expressed in bpm), minute volume (expressed in L/minute) and number of professionals required to perform the chest verticalization protocols. Tidal volume and respiratory rate data were collected directly from the mechanical ventilator monitor immediately at end 30 minutes of each vertical position (as well as in the 3 moments in bed). The measurements were standardized. We followed the proposal by Conti et al.^(12)^

The safety of the interventions was assessed by monitoring the occurrence of following adverse events: hypertension (defined as a systolic blood pressure > 200mmHg or a mean arterial blood pressure > 110mmHg) or hypotension (defined as a mean arterial blood pressure < 65mmHg); a saturation drop (defined as a peripheral oxygen saturation < 88%); tachycardia or bradycardia (defined as a heart rate > 130bpm or < 40bpm, respectively); the onset of arrhythmia; tachypnea or bradypnea (defined as a respiratory rate > 40bpm or < 5bpm, respectively); patient suffering (evidenced by nonverbal signals or gestures); agitation (an RASS score > +1); reduced level of consciousness; becoming physically combative; patient falls; traction or the removal of any devices from the patient; and the interruption of continuous hemodialysis catheter flow. If any adverse events occurred, the protocol was interrupted, and the patient was treated and monitored by the assistant team until clinical stabilization.

### Sample size

Thirty-six subjects were required to achieve a power of 90% to detect an absolute mean difference (MD) in the LUS of 2.0 points (standard deviation - SD, 3.5 points)^(10)^ between the two interventions, with a two-sided alpha level of 0.05. The sample SD was estimated according to the method by Wan et al.^(13)^ using the sample size, median and interquartile range as estimates. The sample SD of each of the groups was estimated, and the average of both was calculated. The base study for such calculations was that of Soummer et al.^(10)^ We predicted that 18 participants would start in the sitting position followed by passive standing and 18 would start with passive standing followed by the sitting position.

### Statistical analysis

Baseline categorical variables are described as absolute and relative frequencies, while baseline quantitative variables are expressed as the mean and SD or median and interquartile range (IQR). Subjects were analyzed according to their randomization group, regardless of the treatment they received. Data distribution was evaluated using graphical analysis and the Shapiro‒Wilk test. Paired Student’s t test was used to compare the primary outcome between the two interventions and perform sensitivity analysis. The Friedman test was used to compare the 5 time points. For the secondary outcomes, categorical outcomes were assessed using McNemar’s test, symmetrical continuous outcomes were evaluated using paired Student’s t test, and continuous asymmetrical outcomes were assessed using Wilcoxon’s signed-rank test. Analyses were performed using R software,^(14)^ version 3.6.3, and a significance level of 5% was set for all analyses.

## RESULTS

### Description of the population

**Figure 1:**
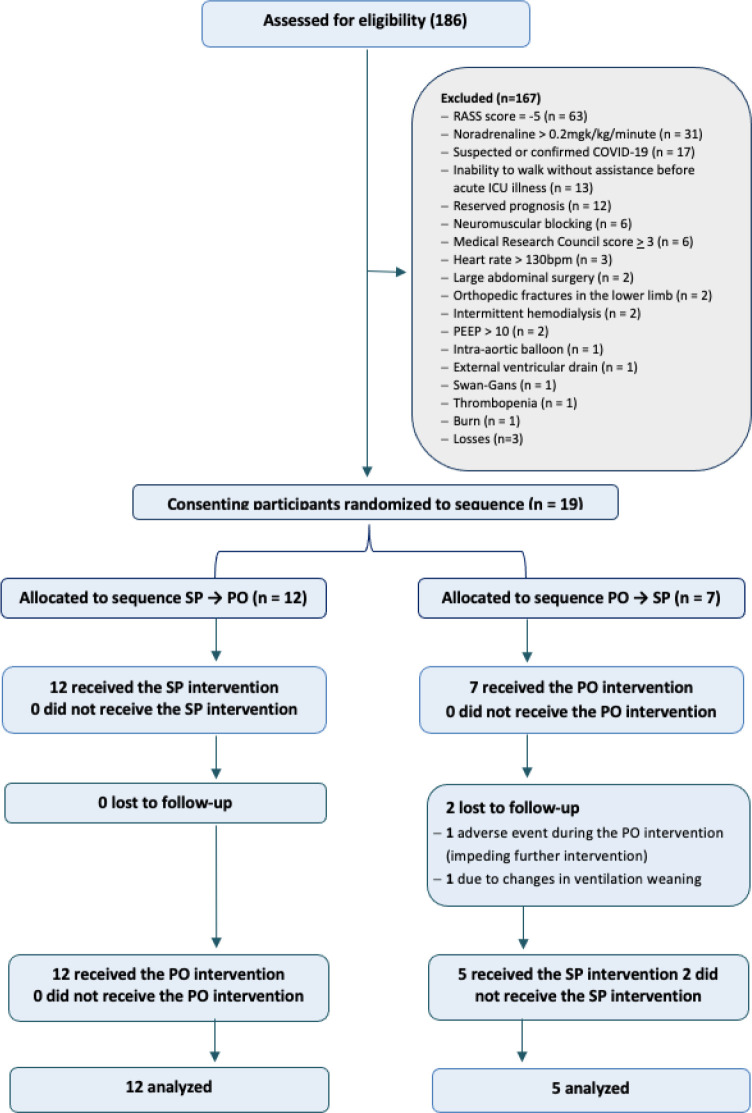
Enrollment, randomization, and follow-up regarding the effect of vertical positioning on lung aeration.

The first subject was enrolled, randomized, and assessed on January 13, 2020; the last subject was screened on July 22, 2020 (Figure 1). In this period, 186 patients were screened. One hundred sixty-seven individuals were excluded. Therefore, 19 participants were enrolled in the study. Of these participants, 12 started in the sitting position. Two subjects did not complete the study protocol (i.e., did not receive both planned interventions): one due to an adverse event during the first intervention (passive orthostasis) and the other due to changes in the ventilation weaning plan after the first intervention (passive orthostasis). Therefore, 17 subjects completed the entire study protocol, while two completed only the first intervention (passive orthostasis), to which they were randomly assigned. Seven patients started with passive orthostasis on the tilt table, and 12 started with the sitting position.

SP - sitting position; PO - passive orthostasis.

The baseline characteristics of the participants are shown in table 1. The mean age was 73.2 years (SD 13.7 years), 75% were aged ≥ 65 years, and 94.7% were admitted to the ICU for medical reasons. Acute respiratory failure was responsible for the initiation of IMV in 57.9% of cases, the mean duration of IMV before randomization was 4.3 days (SD 1.1 days), the mean Simplified Acute Physiology Score 3 (SAPS 3) was 71.6, and the mean RASS score was -4.

**Table 1:** Baseline characteristics

Characteristics	
Age (years)	73.2 ± 13.7
Male sex	11 (57.8)
Charlson comorbidity index score	3.2 ± 1.7
ICU admission type	
Medical	18 (94.7)
Surgical	1 (5.3)
Duration of IMV (days)	4.3 ± 1.1
Reason for mechanical ventilation	
Acute respiratory failure	11 (57.9)
Hemodynamic instability	1 (5.3)
Decreased level of consciousness	5 (26.3)
Cardiac arrest	2 (10.5)
SAPS-3 score	71.6 ± 10.8
Continuous parenteral sedation	3 (15.7)
RASS score	-4 ± 1.1
Mode of mechanical ventilation	
PSV	13 (68.4)
PCV	6 (31.6)

ICU - intensive care unit; IMV - invasive mechanical ventilation; SAPS-3 - Simplified Acute Physiology Score; RASS - Richmond Agitation-Sedation Scale; PSV - pressure support ventilation; PCV - pressure control ventilation. Results expressed as mean ± standard deviation or n (%).

### Primary outcome

**Figure 2:**
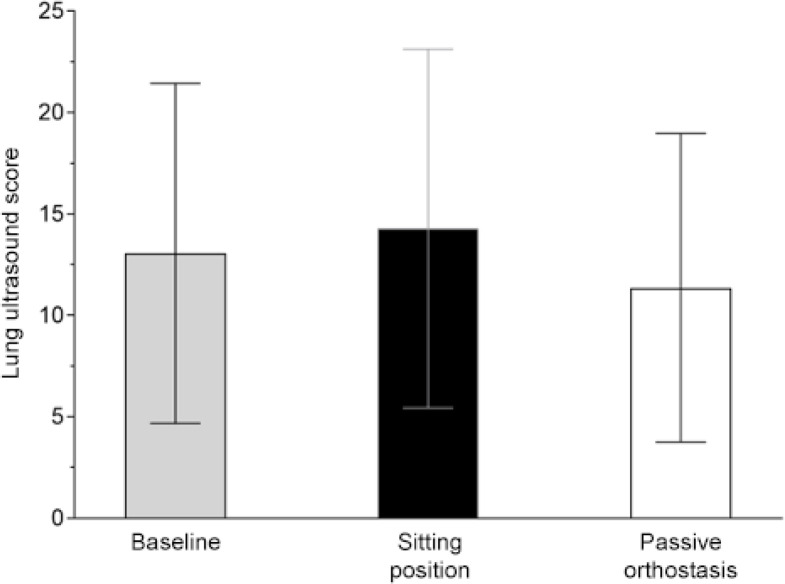
Lung ultrasound score across different verticalization interventions.

**Figure 3:**
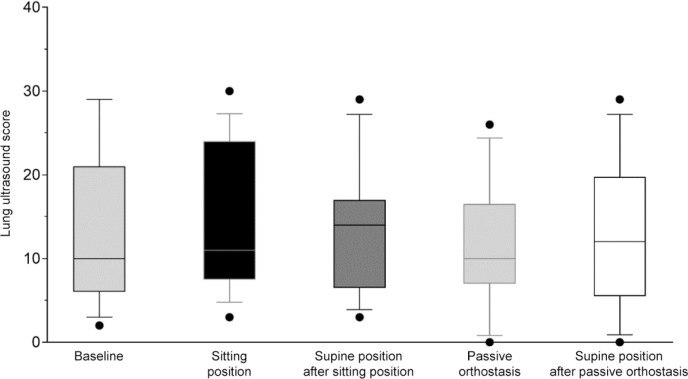
Lung ultrasound scores of patients receiving invasive mechanical ventilation through an endotracheal tube in different postures.

The LUS values across different study time points are shown in figure 2. The mean LUS for the passive orthostasis and sitting positions were 11.0 (SD 8.0) and 13.7 (SD 7.6), respectively (mean difference - MD -2.7; 95% confidence interval - 95%CI -6.1 - 0.71; p = 0.11) (Table 2). No difference in the mean LUS among the 5 time points was observed: baseline: 10 points (6 - 18); sitting position: 12 points (8 - 19); supine position after the sitting position: 14 points (7 - 16); passive orthostasis: 9 points (8 - 12); and supine position after passive orthostasis: 10 points (7 - 14) (p = 0.42) (Figure 3 and Table 3). A *post hoc* sensitivity analysis showed no significant differences between the two study interventions regarding the median variations in the LUS from baseline to the end of the verticalization protocol (passive orthostasis: -1; IQR -5 - 3; sitting position: 0; IQR -1 - 4; p = 0.05).

**Table 2:** Study outcomes

Variables	Passive orthostasis	Sitting position	Mean difference 95%CI	p value
LUS	11 (8.0)	13.7 (7.6)	-2.7 (-6.1 - 0.71)^*^	0.11
RR (bpm)	24.7 (6.1)	24.2 (6.0)	-0.47 (-4.1 - 3.1)^*^	0.78
TV (mL)	436 (395 - 507)	435 (380 - 480)	-8 (-65.0 - 19.0)†	0.47
MV (L/minute)	10.3 (8.7 - 12.5)	11.1 (8.4 - 12.2)	-0.40 (-1.6 - 0.8)†	0.42
Adverse events (patients)	2 (11.8)‡	1 (5.9)‡	-	0.99
Professional staff	3 (3 - 3)†	3 (3 - 3.2)†	-	0.40

95%CI - 95% confidence interval; LUS - Lung ultrasound score; RR - respiratory rate; TV - tidal volume; MV - minute volume. Results expressed as mean and standard deviation (^*^), median and interquartile range (†) or n (%) (‡).

**Table 3:** Lung ultrasound score in different postures

	Baseline	Sitting position	Supine position after sitting position	Passive orthostasis	Supine position after passive orthostasis	p value
LUS	10 (6 - 18)	12 (8 - 19)	14 (7 - 16)	9 (8 - 12)	10 (7 - 14)	0.42

LUS - Lung ultrasound score. Results expressed as median and interquartile range.

The value of the columns indicates the mean, and vertical lines indicate the standard deviation.

The inner horizontal lines of the box plot indicate median; vertical lines, interquartile range and points, most extreme values.

### Secondary outcomes

The median tidal volumes for passive orthostasis and the sitting position were 436mL (IQR 395 - 507) and 435mL (IQR 380 - 480), respectively (p = 0.47). The mean respiratory rates for passive orthostasis and the sitting position were 24.7 (SD 6.1) and 24.2 (SD 6.0), respectively (MD -0.47; 95%CI - 4.1 - 3.1; p = 0.78). The median minute volumes for passive orthostasis and the sitting position were 10.3L/minute (IQR 8.7 - 12.5) and 11.1L/minute (IQR 8.4-12.2), respectively (p = 0.42). There was no difference in the median number of professionals required to perform the passive orthostasis and sitting position protocols (passive orthostasis: 3.0; IQR 3 - 3; sitting position: 3.0; IQR 3 - 3.2; p = 0.40) (Table 2).

At the time the protocol was performed, four adverse events (11%) occurred related to the safety of the interventions. One episode of tachycardia occurred during passive orthostasis and three episodes of hypotension occurred (two during passive orthostasis and one while in the sitting position). The number of patients who experienced adverse events did not differ significantly between protocols (p = 0.99) (Table 2). Thus, there were no serious adverse events reported during the protocols, although early cessation of mobilization due to cardiovascular instability occurred in 3 subjects. None of these patients needed interventions other than the interruption of the protocols.

### Feasibility

There were no significant protocol deviations during the study regarding recruitment procedures, informed consent, intervention administration, or outcome assessment. All included subjects met all the inclusion criteria, and none had any exclusion conditions. Informed consent was obtained from all participants. Although the administration of both interventions was not possible for two subjects, the reason was related to study logistics in only one patient (5%). The washout period was completed for all subjects, and last, there were no missing values for the primary outcome (with the exception of the two participants who did not complete both interventions).

## DISCUSSION

The present crossover randomized clinical trial comparing the effect of passive orthostasis using a tilt-table with a standard sitting position on lung aeration in mechanically ventilated critical care patients found no difference between the interventions. Although no difference in lung aeration was found between the interventions, we cannot rule out the benefits or harms of passive orthostasis using an orthostatic board. This is due to the inclusion of a smaller sample size than needed to accept or refute our hypothesis. Thus, it does not provide sufficient statistical power for definitive conclusions.

The ventilatory benefits of vertical positioning in increasing the end-expiratory lung volume and oxygenation of patients receiving IMV have already been demonstrated.^(15,16)^ For sedated patients receiving IMV in the postoperative period shortly after heart surgery, elevating the headboard up to 30° promotes better lung aeration than 0 or 20° of elevation.^(17)^ Conversely, the vertical position has not always been shown to be better than the horizontal position for oxygenation. A study did not find any difference in lung oxygenation when the supine position was compared to the sitting position for 30 minutes outside the bed by passive transfer.^(18)^ Even when subjects receiving IMV are actively transferred to an armchair and remain seated for 20 minutes, oxygenation may not differ from that in the supine position.^(19)^ Similarly, in our study, the sitting position did not benefit the lung aeration level compared to the orthostatic board. This can occur due to increased intra-abdominal pressure, which impairs ventilation.^(20)^

In the same context of mobilizing patients receiving IMV and assessing lung aeration, Hickmann et al.^(5)^ showed that sitting patients in an armchair and then having them perform exercises improved their lung aeration 20 minutes after exercise. However, only using the sitting position does not increase lung aeration. Likewise, in our study, we could not exclude the benefit of passive orthostasis for lung aeration. Additionally, based on previous studies, although passively, the orthostatic board intervention can lead to an increase in the heart rate and mean arterial pressure.^(21)^ In this sense, greater diaphragmatic activation would increase transpulmonary pressure, leading to better redistribution of air in the lung. Thus, it is essential to highlight the role of exercise in improving pulmonary aeration. On the other hand, as shown in our study, passive orthostasis increases the risk of an adverse event related to postural hypotension.^(22)^ Furthermore, it has been demonstrated that even hypertensive patients can develop hypotension when performing protocols on the tilt table.^(23)^

We believe that the main factors involved in the lack of difference in lung aeration between chest verticalization positions were as follows: first, the low power of the present analysis due to the low number of participants might be associated with type II error. The significant 95%CI found in the primary outcome analysis does not exclude a benefit of passive orthostasis for lung aeration. Second, subjects showed a median LUS of 10 points at baseline. Although the LUS does not have a cutoff point for all populations and situations, Soummer et al.^(10)^ considered lung aeration loss when the LUS was > 14 points at the end of the spontaneous breathing test, which is a good predictor for a high risk of distress after extubation. Likewise, in patients with lung aeration loss, an LUS > 14 points showed a positive correlation with increased respiratory effort, suggesting higher diaphragm demand in response to lung derecruitment.^(24)^ Other studies using the LUS in the IMV weaning process considered a score > 15 points to predict weaning success.^(25)^ An LUS value > 17 points has excellent accuracy in predicting the need for elderly individuals to be admitted to the ICU within 48 hours; otherwise, they will die.^(26)^ Thus, we considered that the subjects in our study did not show significant loss of lung aeration. As a result, they responded poorly to specific procedures such as vertical positioning.

It has been shown that having a small number of multiprofessional staff members is a constraint on the mobilization of critically ill patients.^(27)^ In this study, both verticalization protocols needed the same number of professionals to be performed. It was demonstrated that even out-of-bed mobilizations can be performed without the need of many team members.

Our study had a higher rate of adverse events than reported in a large portion of the literature.^(28,29)^ However, the motor level and time to the beginning of interventions, once IMV has been initiated, are factors that need to be considered. For example, in a study by Eggman et al.,^(28)^ it took 11 days on average to transfer subjects in the intervention group to an armchair. Furthermore, Hodgson et al. ^(30)^ showed that only 5% of patients receiving IMV reached orthostasis with the maximum level of mobilization. In our study, subjects took part in protocols with high mobilization levels after four days on IMV on average. Such procedure heterogeneity, as confirmed by a meta-analysis,^(31)^ may explain the differences between the rates of adverse events.

To the best of our knowledge, this is the first study to evaluate lung aeration in different vertical positions by using the LUS in patients receiving IMV. Studies using the LUS during vertical positioning in patients receiving other ventilation support, such as high-flow nasal cannula (HFNC), noninvasive ventilation (NIV) or IMV by tracheostomy, and evaluating the need for ventilatory support due to pulmonary or extrapulmonary causes may bring new knowledge to clinical practice.

Some limitations must be considered. First, just over 50% of the sample target was met; however, the early interruption of the study was necessary due to the health reality imposed by the COVID-19 pandemic. Second, assessment blinding was not performed, and some evaluators were involved with the study, which may have led to measurement bias. Third, in addition to the small sample, the study was limited to 2 hospitals, which may limit the external validity in other contexts. Fourth, the study design may have led to a carry-over effect. Fifth, we cannot rule out temporal alterations in the patients’ conditions, inherent to the clinical practice of the intensive care environment.

## CONCLUSION

Considering the findings, our study does not allow us to draw generalized conclusions. Even so, we speculate that the verticalization of the chest performed through sitting and passive orthostasis positioning does not generate changes in lung aeration, as assessed by ultrasound. We emphasize that such findings need to be confirmed by a study with a larger population sample.
